# Patient-reported quality-of-life outcomes in relation to provider-assessed adverse events during head and neck radiotherapy

**DOI:** 10.1186/s41687-020-00227-4

**Published:** 2020-07-16

**Authors:** Joshua R. Niska, Cameron S. Thorpe, Michele Y. Halyard, Angelina D. Tan, Pamela J. Atherton, Amylou C. Dueck, Samir H. Patel, Jeff A. Sloan

**Affiliations:** 1 Department of Radiation Oncology, Mayo Clinic, Phoenix, Arizona USA; 2 Division of Biomedical Statistics and Informatics, Mayo Clinic, Rochester, Minnesota USA; 3 Department of Health Sciences Research, Mayo Clinic, Scottsdale, Arizona USA

**Keywords:** Patient reported outcome measures, Quality-of-life, QOL, Radiation oncology, Head and neck neoplasms, Cancer, Radiation therapy, Radiotherapy, RT

## Abstract

**Purpose:**

To assess the relationship between patient-reported quality-of-life (QOL) outcomes and provider-assessed adverse events (AEs) during head-and-neck (H&N) radiotherapy (RT).

**Methods:**

Sixty-five patients undergoing H&N RT prospectively completed 12-domain linear analogue self-assessments (LASA) at baseline, before biweekly appointments, and at last week of RT. At the same time points, provider-assessed AEs were graded using Common Terminology Criteria for Adverse Events v4.0. LASA scores were stratified by maximum-grade AE and analyzed using Kruskal-Wallis methodology. Agreement between LASA scores and maximum-grade AE was assessed using Bland-Altman analysis.

**Results:**

Patient-reported QOL outcomes showed clinically meaningful decreases in most domains, predominantly fatigue (77.8% of patients), social activity (75.4%), and overall QOL (74.2%). Provider-assessed AEs showed 100% grade 2 AE, 35.4% grade 3 AE, and 3.1% grade 4 AE. At baseline, patients with higher grade AEs reported worse physical well-being (WB) (*P* = .04). At week 1, the following QOL domains were worse for patients with higher grade AEs: overall QOL (*P* = .03), mental WB (*P* = .02), and physical WB (*P* = .03). Bland-Altman analysis showed that QOL scores were relatively worse than AE burden at baseline and relatively better at RT completion.

**Conclusions:**

Worse QOL was associated with higher-grade AEs at baseline and early in RT. The impact of AEs on QOL appears to lessen with time. Patient-reported QOL outcomes and provider-assessed AEs provide complementary information.

## Introduction

Patient-reported outcomes (PROs) can be used to monitor quality-of-life (QOL) in real time during cancer treatment. The use of such data unveils otherwise underappreciated problems, results in improved health-related QOL, and facilitates patient-physician communication, all while not prolonging patient encounters [1–4]. Better patient-reported overall QOL has been shown to independently predict improved overall survival for a variety of cancers [5, 6]. Moreover, simply monitoring patient-reported symptoms may improve outcomes. For example, a recent study at Memorial Sloan Kettering Cancer Center, overall survival improved for patients with metastatic cancer who were randomized to longitudinal patient-reported symptom monitoring compared with patients who received usual care (HR, 0.83; *P* = .04) [7]. Given these demonstrated benefits, clinicians [8], federal agencies [9], and international organizations [10] have increasingly promoted the use of PROs in cancer care.

Patients with head-and-neck (H&N) cancer experience well-documented diminished QOL [11–13] and substantial adverse events (AEs) during radiotherapy (RT), with or without chemotherapy [11, 13, 14]. It may seem obvious that QOL should decline with increasingly severe AEs. However, the relationship between patient-reported QOL and AEs is poorly defined. In a pooled analysis of 6 lung cancer trials, patient-reported QOL and *patient-reported* AEs had moderate agreement [15]. In a similar pooled analysis of 12 lung cancer trials, patient-reported QOL and *provider-assessed* AEs had low agreement [16]. To our knowledge, the relationship between patient-reported QOL and AEs has not yet been evaluated in H&N cancer. Given the acute challenges patients face during RT for H&N cancer, understanding QOL and AEs over the course of their treatment is of interest.

Real-time, web-based electronic PROs (ePROs) have proven feasible in outpatient medical oncology [17] and radiation oncology [11] settings. RT provides a unique opportunity to monitor multiple time points during treatment, given that patients are treated daily for several weeks and assessed by providers at least weekly. Our group has previously used ePROs to characterize QOL changes and the burden of AEs at multiple time points during H&N RT [11]. While our prior work independently characterized patient-reported QOL and provider-assessed AEs, we perceived a link between the 2 end points. We hypothesized that diminished patient-reported QOL would be associated with increasingly severe provider-assessed AEs during H&N RT. To address this question, we compared 12 patient-reported QOL domains with provider-assessed AE severity across 5 time points during RT for the subset of 65 patients with H&N cancer enrolled in a previously completed prospective trial approved by the institutional review board.

## Methods and materials

Study design, study measures, prospective data collection, and retrospective data collection have been previously described [11]. Briefly, patients from the previous prospective trial were included in this study if they had undergone curative-intent RT for nonmetastatic H&N cancer. At baseline, before biweekly appointments, and at last week of RT, patients prospectively completed electronic, real-time linear analogue self-assessments (LASA) to assess 12 domains: overall QOL, mental well-being (WB), physical WB, emotional WB, social activity, spiritual WB, pain frequency, pain severity, fatigue level, level of support, financial concerns, and legal concerns [18, 19]. Patient, disease, and treatment characteristics were retrospectively collected from electronic health records. Provider-assessed AEs were retrospectively collected for the same time points as the prospective patient-reported LASA data and graded using Common Terminology Criteria for Adverse Events (CTCAE). CTCAE Version 4.0 includes 790 distinct adverse events that are not necessarily symptom-based (National Cancer Institute, Version 4.0, May 28, 2009).

Scores in each LASA domain were transformed to a 0- to 100- point scale, with 0 being worst and 100 being best. Changes between time points were calculated. A change of 10 or more points on the 0- to 100-point scale was considered clinically meaningful [6]. At each time point, patients were categorized by maximum-grade AE. To match the 0- to 100-point LASA scale, AE grade was transformed to a 0- to 100-point scale (grade 0 = 100, 1 = 80, 2 = 60, 3 = 40, 4 = 20, and 5 = 0). LASA scores were compared to maximum-grade AE using Kruskal-Wallis methodology. Bland-Altman analysis was used to assess agreement between LASA scores and maximum-grade AE [15] Spearman correlation coefficients were used to assess the size of correlations using the criteria published by Cohen: low correlation, 0.10 to 0.29; moderate correlation, 0.30 to 0.49; and high correlation, > 0.50 [20]. All hypothesis testing was completed using 2-sided alternative hypothesis and 5% type I error.

## Results

**Fig. 1:**
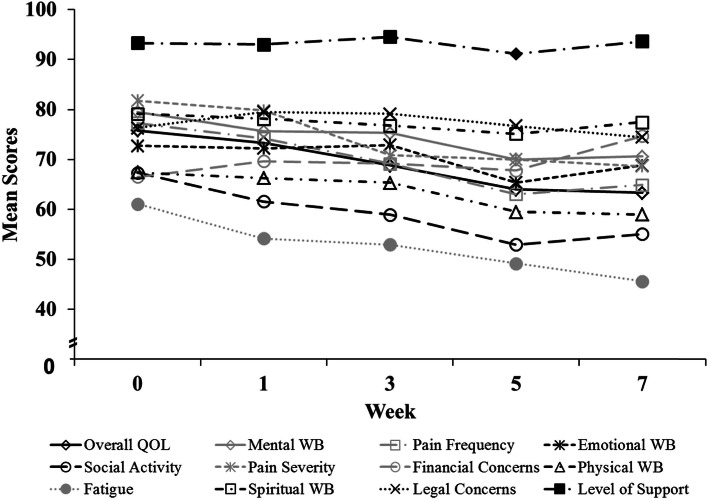
Average LASA scores from baseline to RT completion (0 = Low QOL; 100 = High QOL). Abbreviations: LASA, linear analog self-assessment; QOL, quality of life; WB, well-being. Previously published data [Niska 2017]

Table 1 presents patient, disease, and treatment characteristics for the 65 patients who met the inclusion criteria. Figure 1 displays mean LASA scores across the 12 LASA domains at each time point over the course of RT [11]. Most patients reported clinically meaningful decreases (≥10 points on the 0–100 scale) at some point during RT in the following QOL domains: overall QOL, mental WB, physical WB, emotional WB, social activity, spiritual WB, pain frequency, pain severity, and fatigue level. The QOL domains with the most widespread, clinically meaningful decrease were fatigue (77.8% of patients), social activity (75.4%), and overall QOL (74.2%). At end of RT (week 7), mean scores in the following QOL domains were worse than baseline: overall QOL, mental WB, physical WB, emotional WB, social activity, pain frequency, pain severity, and fatigue level. A more detailed description of the ePROs for QOL has been previously published [11].

**Table 1:** Patient, disease, and treatment characteristics (*N* = 65)

Age	Mean (SD)	65.3 (12.6)			No. (%)
			**Staging**	Stage I	6 (9.2)
**Radiation Dose**	Median (Range) in Gy	60 (50–70)		Stage II	9 (13.8)
				Stage III	10 (15.4)
		No. (%)		Stage IV	39 (60.0)
**Sex**	Male	52 (80.0)		Recurrent	12 (18.5)
	Female	13 (20.0)			
			**Primary Site**	Oral cavity/oropharynx	28 (43.1)
**Race**	White	60 (92.3)		Larynx	8 (12.3)
	Black	2 (3.1)		Salivary gland	7 (10.8)
	Asian	1 (1.5)		Skin	6 (9.2)
	Pacific Islander	1 (1.5)		Nasopharynx	5 (7.7)
	AI/AN	1 (1.5)		Nasal cavity/paranasal sinus	4 (6.2)
				Other	7 (10.8)
**Treatment Type**	Adjuvant CCRT	21 (32.3)			
	Adjuvant RT	20 (30.8)	**Histology**	Squamous cell carcinoma	51 (78.5)
	Definitive CCRT	19 (29.2)		Spindle cell carcinoma	2 (3.1)
	Definitive RT	5 (7.7)		Papillary thyroid carcinoma	2 (3.1)
				Melanoma	2 (3.1)
**Chemotherapy**	Cisplatin	25 (38.5)		Merkel cell carcinoma	2 (3.1)
	Cetuximab	8 (12.3)		Adenocarcinoma	2 (3.1)
	Carboplatin	5 (7.7)		Other	4 (6.2)
	Other	2 (3.1)			

Abbreviations: AI/AN, American Indian/Alaska Native; CCRT, concurrent chemoradiotherapy; RT, radiotherapy

Note: Race was self-identified. Percentages do not add to 100 because of rounding

**Fig. 2:**
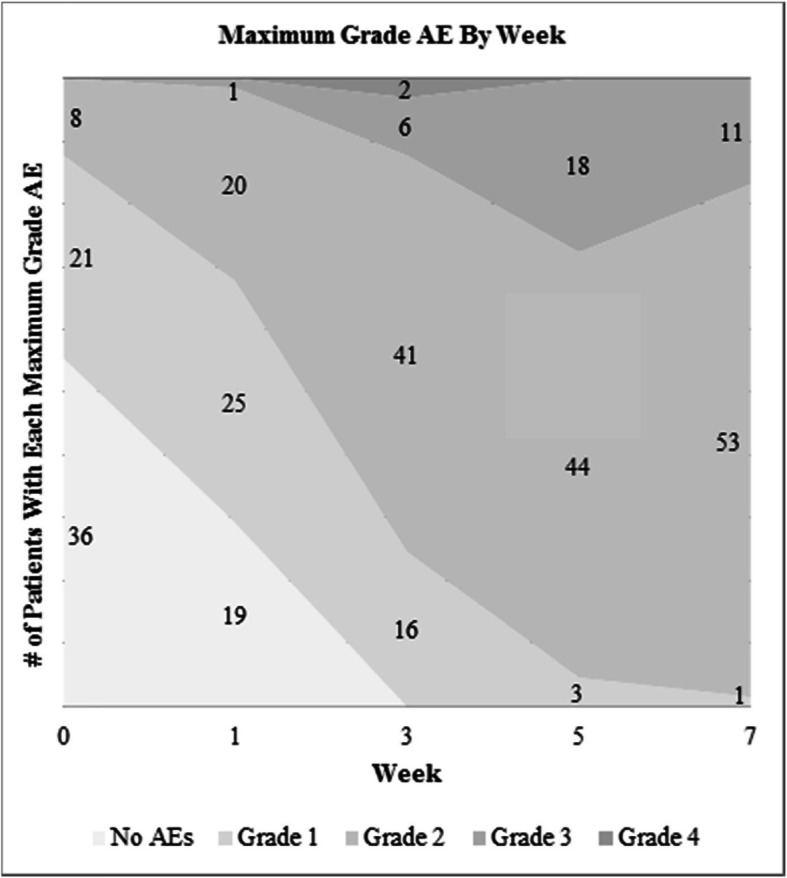
Distribution of maximum grade AE by week. For reference, maximum grade AE distribution at week 3 was grade 1: 16 patients; grade 2: 41 patients; grade 3: 6 patients; grade 4: 2 patients

Figure 2 shows the distribution of maximum-grade AEs at each time point during RT. The area of shading for a given AE grade is proportional to the number of patients with that maximum-grade AE at that time point. The corresponding raw number of patients experiencing a given maximum-grade AE at each time point is also shown. For reference, at week 3, all patients had an AE; 16 patients had maximum grade 1 AE; 41 patients, maximum grade 2 AE; 6 patients, maximum grade 3 AE; and 2 patients, maximum grade 4 AE. Every patient experienced at least grade 2 AE during RT, 35.4% at least grade 3, and 3.1% grade 4. All but 1 patient had at least a grade 2 AE at RT completion. The incidence of specific AEs and their grades over the course of RT have been previously published [11].

For subsequent analyses, the only patient with a grade 3 AE at week 1 was excluded as an outlier, and the 2 patients with grade 4 AEs at week 3 were excluded due to missing data. Table 2 shows the week-by-week mean LASA score for each of the 12 QOL domains. QOL scores are separated into 4 groups: patients experiencing no AE (grade 0), patients experiencing maximum grade 1 AE, patients experiencing maximum grade 2 AE, and patients experiencing maximum grade 3 AE. *P* values are provided to indicate whether patient-reported QOL was significantly different for patients experiencing different grades of AEs. Over the course of RT, multiple QOL domains followed the same general trend regarding maximum grade AE: over the first 3 time points, patients with only grade 1 AEs reported better mean QOL scores, while over the last 2 time points this trend was no longer evident. This pattern was consistent across overall QOL, mental WB, physical WB, emotional WB, social activity, spiritual WB, pain frequency, pain severity, fatigue, and level of support. The only statistically significant QOL differences by maximum-grade AE occurred at baseline (physical WB) and week 1 (overall QOL, mental WB, physical WB). Of note, each domain that was statistically significant (bold in Table 2) also averaged a clinically meaningful change in LASA score (≥10 points on the 0–100 scale). In weeks 3, 5, and 7, QOL scores showed no clear relation to maximum-grade AE.

**Table 2:** QOL Scores by Maximum Grade Adverse Event Per Week

		Grade 0	Grade 1	Grade 2	Grade 3				Grade 0	Grade 1	Grade 2	Grade 3	
	Week	Mean (SD)	Mean (SD)	Mean (SD)	Mean (SD)	*P*-value		Week	Mean (SD)	Mean (SD)	Mean (SD)	Mean (SD)	P-value
**Overall QOL**	0	78.8 (20.0)	75.7 (16.3)	61.4 (24.1)		0.15	**Pain Frequency**	0	84.0 (18.7)	74.3 (23.8)	52.9 (39.9)		0.09
	1	**81.7 (20.4)**	**74.6 (24.1)**	**64.5 (20.1)**		**0.03**		1	72.4 (34.0)	75.8 (28.3)	74.0 (23.5)		0.81
	3		79.3 (16.7)	64.7 (21.4)	68.0 (32.7)	0.09		3		78.0 (21.1)	65.0 (23.0)	74.0 (20.7)	0.15
	5		63.3 (15.3)	65.2 (21.4)	60.7 (22.8)	0.72		5		50.0 (28.3)	64.1 (24.2)	61.9 (25.9)	0.69
	7			62.1 (20.0)	77.5 (20.6)	0.16		7			65.7 (26.5)	55.0 (26.5)	0.35
**Mental WB**	0	80.6 (20.3)	80.5 (14.7)	71.4 (21.2)		0.43	**Pain Severity**	0	86.3 (15.0)	78.6 (17.7)	68.6 (34.4)		0.22
	1	**81.7 (20.9)**	**79.2 (17.4)**	**66.0 (19.0)**		**0.02**		1	86.3 (17.5)	77.9 (24.8)	77.0 (22.0)		0.32
	3		78.7 (16.4)	73.7 (18.9)	78.0 (29.5)	0.49		3		80.0 (15.1)	67.9 (21.3)	66.0 (28.8)	0.20
	5		63.3 (5.8)	71.2 (22.0)	68.1 (23.4)	0.57		5		60.0 (28.3)	71.0 (21.0)	68.8 (20.9)	0.67
	7			69.4 (18.8)	85.0 (12.9)	0.10		7			69.4 (23.2)	60.0 (18.3)	0.34
**Physical WB**	0	**69.4 (19.1)**	**70.5 (17.2)**	**48.6 (19.5)**		**0.04**	**Fatigue**	0	66.0 (27.1)	55.7 (26.9)	52.9 (27.5)		0.22
	1	**71.1 (24.7)**	**71.3 (23.3)**	**56.0 (17.9)**		**0.03**		1	60.6 (25.3)	59.2 (26.2)	42.5 (17.4)		0.08
	3		70.7 (17.5)	63.7 (20.5)	62.0 (25.9)	0.58		3		57.3 (27.9)	50.3 (25.8)	60.0 (15.8)	0.50
	5		60.0 (20.0)	61.5 (21.9)	54.4 (25.8)	0.62		5		33.3 (11.5)	49.8 (24.2)	50.6 (18.4)	0.36
	7			57.9 (19.7)	72.5 (17.1)	0.16		7			44.7 (23.3)	60.0 (34.6)	0.48
**Emotional WB**	0	75.6 (20.8)	72.9 (17.1)	58.6 (23.4)		0.15	**Level of Support**	0	94.3 (11.4)	94.3 (12.1)	85.7 (29.9)		0.94
	1	77.2 (22.7)	72.9 (21.8)	67.0 (20.3)		0.22		1	93.9 (16.1)	94.6 (7.8)	90.5 (15.4)		0.31
	3		78.7 (13.0)	70.3 (22.1)	76.0 (33.6)	0.44		3		95.0 (8.5)	94.5 (10.3)	94.0 (13.4)	0.93
	5		66.7 (11.5)	65.0 (21.6)	66.3 (24.7)	0.94		5		100.0 (0.0)	91.2 (15.0)	89.4 (15.3)	0.30
	7			68.1 (19.9)	77.5 (20.6)	0.34		7			93.5 (11.4)	96.7 (5.8)	0.74
**Social Activity**	0	71.2 (25.9)	67.5 (20.5)	48.6 (38.0)		0.23	**Financial Concerns**	0	64.9 (31.9)	63.8 (35.1)	82.9 (37.3)		0.21
	1	63.9 (31.5)	65.0 (27.0)	55.5 (21.9)		0.41		1	67.2 (33.7)	66.7 (30.3)	75.5 (26.8)		0.68
	3		71.3 (21.0)	54.5 (25.9)	56.0 (31.3)	0.11		3		64.7 (34.2)	70.0 (31.5)	76.0 (37.8)	0.69
	5		66.7 (25.2)	54.5 (25.9)	46.3 (27.5)	0.40		5		93.3 (11.5)	69.8 (32.2)	58.1 (32.3)	0.10
	7			54.5 (23.0)	62.5 (33.0)	0.51		7			75.7 (26.1)	60.0 (52.9)	0.72
**Spiritual WB**	0	81.5 (20.9)	78.0 (17.9)	70.0 (26.8)		0.42	**Legal Concerns**	0	74.6 (34.0)	72.4 (33.2)	97.1 (7.6)		0.07
	1	82.8 (17.4)	77.1 (25.6)	74.7 (25.0)		0.82		1	84.4 (25.0)	77.1 (35.1)	78.0 (32.5)		0.77
	3		78.0 (17.4)	76.5 (20.7)	75.0 (37.9)	0.89		3		66.0 (37.0)	82.9 (28.2)	90.0 (22.4)	0.07
	5		90.0 (10.0)	75.3 (21.7)	72.0 (26.8)	0.49		5		86.7 (23.1)	78.8 (31.3)	69.4 (34.7)	0.52
	7			76.7 (20.9)	85.0 (19.1)	0.40		7			76.9 (32.1)	47.5 (37.7)	0.15

Analyzed using Kruskal-Wallis methodology

The only patient with grade 3 adverse event at week 1 was excluded

The only two patients with grade 4 adverse events were excluded

**Fig. 3:**
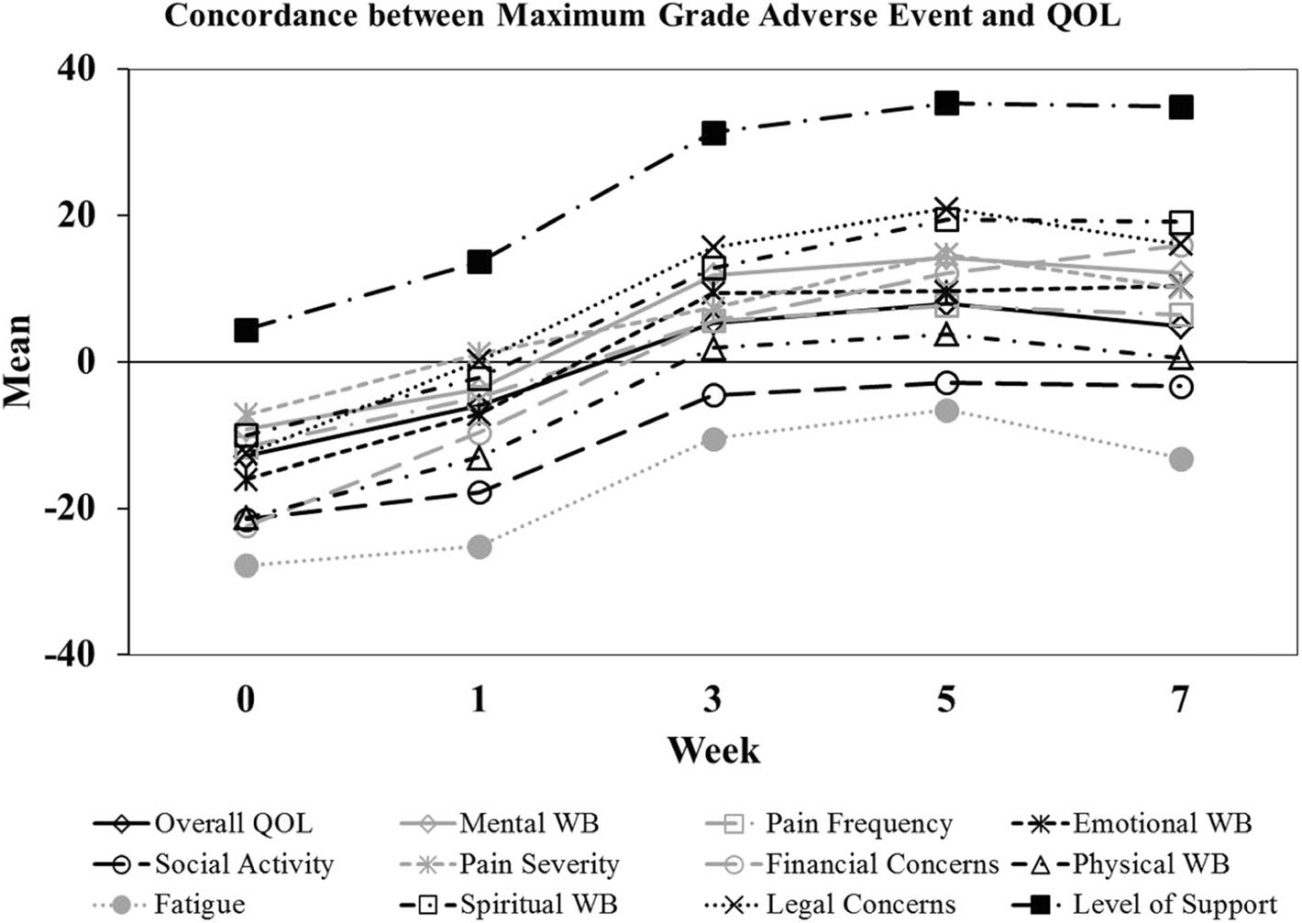
Bland-Altman analysis to assess agreement between AE grade and LASA score at each time point. Both were transformed to 0 to 100 scale, and LASA score was subtracted from AE score

Where QOL score was statistically different by maximum-grade AE, Spearman correlation coefficients were 0.34 for week 1 overall QOL (moderate correlation), 0.35 for week 1 mental WB (moderate), 0.18 for baseline physical WB (low), and 0.28 for week 1 physical WB (low). Bland-Altman analysis, used to assess agreement between AE grade and LASA score at each time point, revealed differential agreement over the course of RT (presented as mean LASA score minus mean AE score at each time point, Fig. 3). After transforming to 0 to 100 scale, mean AE score minus mean LASA score was lower at baseline than week 7 across all QOL domains.

## Discussion

To our knowledge, our study provides the first assessment of the relationship between patient-reported QOL and provider-assessed AEs during H&N RT. Although H&N RT can impact all aspects of QOL, fatigue, social activity, and overall QOL are most commonly affected. All patients experienced grade 2 AE, many experienced grade 3 AE, and few experienced grade 4 AE. Excluding 1 outlier, grade 3 AEs began in week 3 and peaked before end of RT. At baseline and early in RT, clinically meaningful decreases in QOL were associated with more severe AEs, with low to moderate correlation. As time passed, QOL lost its correlation with AE severity.

Although QOL worsened and AE severity increased over the course of RT, correlation between them was only present at baseline and week 1. Toxicities at these early time points are more likely to be caused by prior treatment (eg, surgery or chemotherapy) or by the disease itself, and less likely to be caused by RT. Patient anticipation of upcoming RT may also play a role. Of note, the toxicities that correlated with clinically meaningful decreases in QOL were all grade 2. Understandably, clinicians often focus on grade 3 or higher AEs late in RT or after RT completion. Nonetheless, early grade 2 AEs appear to be negatively associated with patient-reported QOL and should be proactively addressed.

As any clinician can attest, patient concerns and provider concerns are often incongruent. For example, in a phase II trial of metastatic prostate cancer, *physician-reported* symptoms were only 47% sensitive and 68% specific for *patient-reported* symptoms [21]. Prior studies in lung cancer have shown low correlation between patient-reported QOL and *provider-assessed* AEs [16] but moderate correlation between patient-reported QOL and *patient-reported* AEs [15]. Furthermore, agreement between *provider-assessed* AEs and *patient-reported* AEs has been shown to decline throughout H&N RT [22]. To our knowledge, there are no reports in the literature comparing patient-reported QOL and provider-assessed AEs for patients with H&N cancer. We showed low to moderate correlation between patient-reported QOL and provider-assessed AEs at early time points for patients having H&N RT. No correlation was present at later time points. This rapid decline in correlation between patient-reported QOL and provider-assessed AEs may be due to a number of factors: patients may become accustomed to mounting treatment-related toxicities, providers may be adequately addressing patient concerns, or perhaps QOL becomes truly independent of AEs as patients become more resilient.

Our study has several limitations. This was an unplanned secondary analysis of data from a prospective clinical trial. As such, our findings should be treated as hypothesis-generating and need to be confirmed by future prospective studies. The demographics of our institution may limit the generalizability of our results (92.3% of patients included in the study were white). With only 65 patients, statistical power was limited. Two patients with maximum grade 4 AE at week 3 were excluded due to missing data. Furthermore, to minimize the effect of a single outlier on our analysis, we excluded the 1 patient with maximum grade 3 AE at week 1. We had relatively few AEs ≥ grade 3. However, early grade 2 AEs did predict QOL decrement and may portend future grade 3 AEs. In a larger sample with more high-grade AEs, we may indeed find association between ≥ grade 3 AEs and QOL. Furthermore, using a QOL instrument specific to H&N cancer may show greater association with AEs. Additionally, we only captured AEs and QOL *during* RT. In the pooled analysis of lung cancer trials by Huschka et al., moderate agreement between ≥ grade 3 AEs and QOL was seen *after* RT [15]. In the future, capturing data both during and after RT may allow for better characterization of the relationship between patient-reported QOL and provider-assessed AEs. Moreover, our study did not include patient-reported AEs, which are emerging as an important complementary end point for future trials [23].

## Conclusions

During H&N RT, patient-reported QOL has a complex relationship with provider-assessed AEs. At least initially, grade 2 AEs matter to patients: clinically meaningful decreases in overall QOL, mental WB, and physical WB have low to moderate correlation with grade 2 AEs at baseline and early in RT. Over the course of RT, QOL worsens, and the burden of AEs increases. However, patient-reported QOL does not correlate with provider-assessed AEs after week 1. Therefore, patient-reported QOL complements *provider-assessed* AEs during H&N RT. The role of *patient-reported* AEs continues to evolve. Going forward, clinical trials that include patient-reported QOL, patient-reported AEs, and provider-assessed AEs may ultimately improve cancer care.

## Data Availability

We have full control over the primary data and will allow it to be reviewed if requested.

## Abbreviations

AE: Adverse event
CTCAE: Common terminology criteria for adverse events
ePROs: Electronic patient-reported outcomes
H&N: Head and neck
LASA: Linear analog self-assessment
PROs: Patient-reported outcomes
QOL: Quality-of-life
RT: Radiotherapy
WB: Well-being
